# Evaluation of an mHealth Intervention (Growin’ Up Healthy Jarjums) Designed With and for Aboriginal and Torres Strait Islander Mothers: Engagement and Acceptability Study

**DOI:** 10.2196/43673

**Published:** 2023-05-26

**Authors:** Sarah Jane Perkes, Belinda Huntriss, Noelene Skinner, Bernise Leece, Rosie Dobson, Joerg Mattes, Kerry Hall, Billie Bonevski

**Affiliations:** 1 Flinders Health and Medical Research Institute College of Medicine and Public Health Flinders University Bedford Park, South Australia Australia; 2 School of Medicine and Public Health College of Health, Medicine and Wellbeing University of Newcastle New South Wales Australia; 3 National Institute for Health Innovation University of Auckland Auckland New Zealand; 4 First Peoples Health Unit, Griffith University Southport Australia

**Keywords:** mobile health, mHealth, co-design, Aboriginal and Torres Strait Islander, mother, baby, young children, mobile phone

## Abstract

**Background:**

Aboriginal and Torres Strait Islander women have access to and interest in mobile health (mHealth), although few culturally relevant, evidence-based mHealth programs are available. We codeveloped an mHealth program in New South Wales with Aboriginal and Torres Strait Islander women, focusing on women’s and children's health and well-being.

**Objective:**

This study aims to assess the engagement with and acceptability of the Growin’ Up Healthy Jarjums program among mothers caring for Aboriginal and Torres Strait Islander children aged <5 years and assess the acceptability of the program among professionals.

**Methods:**

Women were given access to Growin’ Up Healthy Jarjums—a web-based application, a Facebook (Meta Platforms, Inc) page, and SMS text messages—for 4 weeks. Short videos of health professionals presenting health information were tested within the application and on the Facebook page. Engagement with the application was examined through the number of log-ins, page views, and links used on the application. Engagement with the Facebook page was examined through likes, follows, comments, and the reach of posts. Engagement with the SMS text messages was examined through the number of mothers who opted out, and engagement with the videos was examined through the number of plays and videos watched and duration of the video watched. The acceptability of the program was examined through posttest interviews with mothers and focus groups with professionals.

**Results:**

A total of 47 participants joined the study (n=41, 87%, mothers and n=6, 13%, health professionals). Interviews were completed by 78% (32/41) of the women and 100% (6/6) health professionals. Of the 41 mothers, 31 (76%) women accessed the application, 13 (42%) scrolled the main page only, and 18 (58%) clicked on other pages. There were 48 plays and 6 completions of the 12 videos. The Facebook page received 49 page likes and 51 followers. The post with the most reach was a supportive and affirming cultural post. No participants opted out of the SMS text messages. Almost all mothers (30/32, 94%) reported that Growin’ Up Healthy Jarjums was useful, and all mothers reported that the program was culturally appropriate and easy to use. Of the 32 mothers, 6 (19%) mothers reported technical problems with accessing the application. Moreover, 44% (14/32) of mothers suggested improvements to the application. All the women reported that they would recommend the program to other families.

**Conclusions:**

This study demonstrated that the Growin’ Up Healthy Jarjums program was perceived useful and culturally appropriate. SMS text messages had the highest engagement, followed by the Facebook page and then the application. This study identified areas for technical and engagement-related improvements to the application. A trial is needed to assess the effectiveness of the Growin’ Up Healthy Jarjums program at improving health outcomes.

## Introduction

### Background

Aboriginal and Torres Strait Islander people are the oldest surviving culture in the world [1]. The health of Aboriginal and Torres Strait Islander people changed significantly upon colonization and has continued to be disrupted by subsequent policies [2]. Improving the health and lives of Aboriginal and Torres Strait Islander people is a national priority. Mothers and babies receiving the best possible care and support for a good start to life is 1 of the 12 health priorities of the National Aboriginal and Torres Strait Islander Health Plan 2013-2023 [3]. Providing access to culturally responsive health information and services is an important strategy for achieving this goal [3].

Improving health literacy provides a foundation for individuals and communities to take action to improve their own health [4]. There is limited evidence on effective health literacy programs for Aboriginal and Torres Strait Islander people [5]. A systematic review examining interventions for improving health literacy among Aboriginal and Torres Strait Islander people included 5 studies with the following interventions: exercise classes, nutrition and cooking workshops, discussions and role plays, presentations, other learning activities, incentives, and reduction in the cost of fresh and frozen produce and low-sugar beverages and education at the point of sale [5]. All the included studies demonstrated statistically significant improvements in at least 1 health literacy–related outcome measure, although it should be noted that study quality was compromised because of small sample sizes and poor attendance [5]. More rigorous trials are needed on health literacy programs designed and implemented by Aboriginal and Torres Strait Islander people for Aboriginal and Torres Strait Islander people*.*

An array of mobile technologies is available to find, share, and generate health information [6]. The major benefit of mobile health (mHealth) is its ability to reach a large number of consumers, including those who cannot attend health services. Aboriginal and Torres Strait Islander women have a high interest in using mHealth [7] but have different preferences for delivery as well as content. Evidence to date shows that Aboriginal and Torres Strait Islander people are frequent users of Facebook (Meta Platforms, Inc) [8-11] and SMS text messaging [12-14] and report high acceptability of, but low engagement with [15], apps, as is often the case universally [16]. Content that centers on culture and frames positive health messages has greater acceptability [9,10,17]. Furthermore, certain delivery mechanisms may be particularly engaging to mothers. A report on Australian women’s use of digital health found that women caring for infants and young children were more likely than other women to use social media and web-based forums to share and create health information [8], whereas other studies have found SMS text messaging to have high acceptability among mothers [10,11].

Available mHealth programs for Aboriginal and Torres Strait Islander mothers or their children are limited but growing, including an app, a website, and SMS text messaging on infant feeding [18]; SMS text messaging, videos, and multimedia messaging service for otitis media in children [13]; SMS text messaging, a phone call, Facebook, or an email for postpartum blood glucose screening [12]; a prototype app for social and emotional well-being during pregnancy [19]; and a mindfulness app for women and children of all ages [20]. In the gray literature, the authors are aware of the Deadly Tots app and interactive website on child development [21] and Facebook pages such as Stay Strong and Healthy page for health during pregnancy [21] and Yarn and Heal—Our way for Aboriginal women of all ages to connect and yarn [22]. It is important that we seek to advance mHealth solutions developed by and for Aboriginal and Torres Strait Islander women to promote digital inclusion and access to health information, particularly as it is known that cultural minorities are less likely to use mainstream web-based health technologies [6].

In 2019, we co-designed a multimodality mHealth program for Aboriginal and Torres Strait Islander women’s and children’s health [23]. The aim of the program was to improve health literacy and health behaviors as well as increase access to health services. Formative research with 31 women and 11 health professionals took place in 3 communities in New South Wales (NSW) and included focus groups with storyboards, card sorting, and design activities [23]. On the basis of the findings from the formative research, we developed a web-based prototype application, an SMS text message library, and a Facebook page, collectively called the Growin’ Up Healthy Jarjums (an Yugambeh word used on the East Coast of Australia meaning children) program.

Following a formative research phase, subsequent steps to develop and evaluate mHealth interventions include conducting a pilot study, a randomized control trial, and an evaluation of the implementation impact [24]. The purpose of the pilot study stage is to determine acceptability, improve and refine the intervention, and test the content and regimen early in the research process [24]. Refining the intervention is often an iterative process in each research phase and beyond [24]. Continual improvements to mHealth interventions are important, given the constant upgrades to technology and that long-term engagement with mHealth can be difficult to achieve [25]. We used a pilot study design to evaluate the acceptability of and engagement with the Growin’ Up Healthy Jarjums prototype program.

### Objectives

The aims of this study were (1) to assess the engagement with the Growin’ Up Healthy Jarjums program among mothers (or other women) caring for Aboriginal and Torres Strait Islander children aged ≤5 years; (2) to assess the acceptability of the Growin’ Up Healthy Jarjums program among mothers (or other women) caring for Aboriginal and Torres Strait Islander children aged ≤5 years; and (3) to assess the acceptability of the Growin’ Up Healthy Jarjums program among health professionals and early educators.

## Methods

### Project Design

A 4-week pilot study of the Growin’ Up Healthy Jarjums mHealth program was undertaken with Aboriginal and Torres Strait Islander women caring for children aged <5 years. Health professionals and early educators from the participating services provided feedback on the intervention in focus groups. Details on the development of the Growin’ Up Healthy Jarjums mHealth program can be found elsewhere [23]. The Aboriginal Health and Medical Research Council (AH&MRC) Ethical Guidelines: Key Principles (2020) version 2.0 were used to guide the implementation of this pilot study [26].

### Research Team

This research was governed by an Aboriginal advisory board in partnership with Aboriginal organizations (listed in the *Acknowledgments* section) and coled by a Kuku Yalanji and Lama investigator (KH), as well as 2 non-Indigenous investigators (BB and JM). In total, 3 team members were Aboriginal women from (or connected to) the communities where the research took place: a Gumbaynggirr woman (NS), Gomeroi woman in the Kamilaroi Nation (BL), and Worimi woman working in the Awabakal community (BH). The cultural identities of the remaining team members are Macedonian-Australian (BB), German-Australian (JM), Pakeha or European-New Zealand (RD), and European-Australian (SJP). The team has various professional backgrounds: 4 women with Aboriginal lived experience (KH, NS, BL, and BH), a behavioral scientist (BB), a pediatrician and academic (JM), a nurse and public health researcher (KH), an mHealth and public health researcher (RD), an early educator (BH), an Aboriginal health practitioner (NS and BL), and an occupational therapist and PhD candidate (SJP). All team members contributed to the conception of this study. Aboriginal researchers from the participating communities (NS, BH, and BL) led the implementation of the project to support cultural safety.

### Participant Sampling

Women aged ≥16 years who were either mothers or primary carers of Aboriginal or Torres Strait Islander children aged birth to 5 years or were pregnant (≥30 weeks’ gestation), owned or regularly used a smartphone, and had accessed a participating service (an Aboriginal health service or NSW health service) were eligible to participate. Health professionals from the participating health services and early educators from the participating preschools of all cultural identities who worked with women or children were eligible.

### Procedures

This study was conducted remotely from August 2020 to March 2021 using telephone, SMS text messages, and videoconferencing owing to COVID-19 restrictions. Participants were recruited from 3 regional locations in NSW, Australia. A total of 5 Aboriginal organizations (2 Aboriginal health services, 2 Aboriginal preschools, and 1 Aboriginal family and parenting corporation) and 3 NSW health sites participated. In total, 2 Aboriginal researchers (NS and BH) completed most of the recruitment, consent procedures, interviews, and communication with the participants and services in line with the AH&MRC Ethical Guidelines to ensure culturally safe, best practice research procedures *(2.2.3, 2.3.3, 2.5.2, and 3.3.1)* [26]*.*

Women who participated in the co-design phase [23] were contacted via phone and invited to participate in the pilot study. Convenience snowball sampling was also used [27]. The Aboriginal researchers (BH, NS, and BL) used their personal networks to recruit additional participants. The participants were also asked whether they would like to recommend a friend or family member to the study. The participating health services also reached out to potential participants. Potential participants were screened for eligibility when contacted by the researcher via phone. The researcher explained the study and obtained informed consent. The participants were sent an SMS text message with a link to a baseline survey on REDCap (Research Electronic Data Capture; Vanderbilt University) before starting the pilot study. During the 4-week study period, the participants were given access to the intervention (Textbox 1). The participants were sent a link via an SMS text message to access the application, and where possible, the research team contacted the participants to check whether they were able to access the application, explain the use of the application, check whether they were receiving SMS text messages, and explain how to like the Facebook page. The participants were asked to access the application as often as they felt compelled to, that is, there was no required amount of time that women needed to spend on the application or other parts of the program. Following the Facebook page was optional. After 4 weeks, the participants were contacted via telephone for an interview. Semistructured interviews with a mixture of open- and closed-ended questions [28] were conducted by Aboriginal researchers (NS and BH) and a non-Indigenous PhD student (SJP). The interviews were 6 to 25 minutes in length. They were recorded and transcribed, and interview notes were taken as a backup to recordings [28]. The participants were reimbursed with a shopping voucher worth Aus $20 (US $30) at baseline, a shopping voucher worth Aus $10 (US $15) per week for the 4-week pilot study Aus $40 (US $60) in total to cover data use, and a shopping voucher worth Aus $20 (US $30) for participating in the follow-up interview. The interviews were completed between August and September 2020.

**Components of the prototype intervention.**

**Application**

- The application is a central place for users to access all content. The application is primarily for the user who wants in-depth information and has the necessary digital device, internet connection, and literacy skills to access it. The application has four menu screens as follows: (1) home screen, (2) women’s health, (3) children’s health, and (4) contacts. The Facebook (Meta Platforms, Inc) page content feed was embedded into the home screen. The women’s health menu page includes six buttons, one for each of the women’s health modules as follows: (1) smoke-free families, (2) safe drinking, (3) feeling good, (4) women’s business, (5) eating, and (6) exercising. The Jarjum’s Health modules include (1) breathing well; (2) sleeping; (3) milestones; (4) feeding and eating; (5) vaccinations and medicines; and (6) ears, eyes, and teeth. Each topic includes (1) key messages incorporating the perceived threat of illness and benefits of changing health behavior; (2) tips to address the barriers to change through reassurance and credible advice; (3) cues to action, for example, “Each time jarjum sees a nurse or GP ask them to have a quick look in bub’s ears to check if there is any infection”; and (4) links to further information, including information regarding skills and activities such as exercises and healthy recipes to support self-efficacy. The information is presented using small chunks of written information and videos using the same layout in each module.

**Videos**

- A total of 12 videos were developed (1 per topic). The length of the videos ranged from 1 minute and 42 seconds to 5 minutes. The videos included health professionals from the participating sites or contacts of the research team presenting key messages on each health topic. The presenters were given short scripts and encouraged to use their own expertise and experience. The videos were displayed in the application under each topic as well as added to the Facebook feed at least once. The users were able to watch the videos within the application; however, on Facebook, the users were taken to an external Vimeo (Vimeo, Inc) platform to view.

**Facebook page**

- The purpose of the Facebook page was to create community and connection, allow 2-way communication, and use a platform that is highly popular among users. Daily content was added to the Facebook page, including (1) links to reliable health websites, (2) activities for families, (3) weekly competitions, (4) key messages on the health topics listed earlier (written and video), (5) events in the community, and (6) supportive affirmative posts. The page was administrated by 2 Aboriginal team members (NS and BH), who shared posts relevant to their community and region.

**SMS text messaging**

- The SMS text messaging component allowed the users access to health information regardless of their mobile phone type, Wi-Fi access, or digital literacy. The SMS text messaging portion of the program was 1 way (unidirectional). The SMS text messages included two core topics: (1) breathing well and (2) smoke-free families, and the participants chose 3 additional topics (from the topics covered in the application). The women received 1 message per day for 5 days per week for 4 weeks (20 SMS text messages in total).

The professionals who participated in the co-design phase were contacted via phone and invited to participate in the pilot study. Where these professionals were no longer working at the service, other professionals known to the Aboriginal researchers were contacted to participate. A total of 2 focus groups were conducted in February and March 2021. Focus groups [28] were conducted rather than individual interviews as per the professionals’ preference. Consent was initially obtained over the phone and then again in person, videoconference, or email before starting the focus group. A brief survey was conducted at the start of the focus group. The professionals accessed the program during the focus group only (not during the 4-week pilot study). We were interested in the professional’s feedback on the content only, not in how they might engage with the program over 4 weeks, as they were not the target end users. It was important, however, to obtain feedback from professionals who routinely provide health information to mothers, as they may be instrumental to the implementation of the program, if the program is effective. One focus group was conducted in person (as COVID-19 restrictions had been lifted) and another over videoconference. The focus groups were 13 and 21 minutes in length. The professionals were not reimbursed.

### Measures

#### Demographics and Cultural Characteristics

The survey completed by mothers was a 16-item survey including demographic, cultural, and socioeconomic items. The items were selected from a previous study [29], with all items having been tested with Aboriginal and Torres Strait Islander mothers previously. The survey completed by professionals comprised 5 items related to demographic and professional practice characteristics.

#### Engagement

Objective measures are common for measuring the engagement of applications [30]. The user activity metrics collected included the number of log-ins, number of page views, length of page view, and number of links used on the application. We used user activity metrics in combination with interview data to identify user typologies. Data collected for the videos included the number of plays in total and per video, number of videos watched in full (completions), duration watched (in seconds; mean seconds and percentage), and number of unique videos. Data collected for the Facebook page included the number of posts by administrators, number of page likes, number of comments, number of followers, and the reach of posts and videos. Data were collected on topics that the women chose to receive SMS text messages on and the number of women who opted out of receiving SMS text messages. User engagement was evaluated only for the women participants (end users), not for the professionals.

#### Acceptability

An interview schedule was adapted from a previous study on the acceptability of a culturally tailored SMS text messaging program for mothers [31]. The interview schedule included the following topics: usefulness of the program, cultural appropriateness of the program, ease of understanding, appropriateness of the program, relevance of the program, perceived impacts, and suggestions for improvements. A shortened and adapted version of the interview schedule was used with professionals, which included items on usefulness and cultural appropriateness.

### Data Analysis

The interview data were analyzed and summarized using descriptive quantitative analyses including means, SDs, and proportions [32]. Qualitative comments were analyzed using a simple thematic analysis with predetermined codes based on the research areas, for example, cultural appropriateness [28]. One of the researchers (SJP) cleaned and coded the responses for each predetermined code. Then, 3 researchers (SJP, BH, and NS) reflected on and discussed the participant quotes to form a summary statement for each code and select representative quotes.

### Ethics Approval

Human research ethics approval was received from the AH&MRC (1485/19) and University of Newcastle (H-2019-00760).

## Results

### Overview

A total of 47 participants were recruited for the study: 41 (87%) women and 6 (13%) health professionals. The average age of the women was 31 (SD 7.35) years. The women were from 15 different communities; Kamilaroi (12/41, 29%) and Gumbaynggirr (10/41, 24%) were the most common. Almost half of the women (20/41, 49%) in this study had participated in the co-design phase of the project. The demographic characteristics of the participants are presented in Tables 1 and 2.

**Table 1:** Demographic and cultural characteristics of women (n=41).

Characteristics		Values
Age (years), mean (SD; range)		31.54 (7.35; 17-50)
**Participation in the co-design phase, n (%)**		
	Yes	20 (49)
	No	19 (46)
	Not sure	2 (5)
**Indigenous status, n (%)**		
	Aboriginal	34 (83)
	Torres Strait Islander	0 (0)
	Both	0 (0)
	Nonidentified	6 (15)
	Unknown	1 (2)
**Identified with an Indigenous community, n (%)**		
	Yes	25 (61)
	No	5 (12)
	Unknown	11 (27)
Maintain cultural connections at home (yes), n (%)		28 (68)
**Ways of connecting to culture, n (%)**		
	Music or dance	22 (82)
	Storytelling	21 (78)
	Art	20 (74)
	Indigenous television	18 (67)
	Food	12 (44)
	Indigenous internet sites	12 (44)
	Indigenous newspapers	7 (26)
	Traditional medicine	5 (19)
	Indigenous radio	4 (15)
	Other	3 (11)
**Family members from the Stolen Generations^a^, n (%)**		
	Yes	14 (34)
	No	13 (32)
	Unknown	14 (34)
**Education of the mother, n (%)**		
	Did not finish high school	7 (17)
	High school	13 (32)
	Certificate	11 (27)
	Bachelor’s degree	4 (10)
	Diploma	3 (7)
	Postgraduate degree	2 (5)
	Not applicable	1 (2)
Number of people living in household, mean (SD; range)		4.17 (1.72; 1-8)
Number of children (aged <18 years) living in household, mean (SD; range)		2.44 (1.48; 1-6)
**Smoking status of the mother, n (%)**		
	Nonsmoker	31 (76)
	Yes, daily	10 (24)
	Yes, at least once a week	0 (0)
	Yes, less often than once a week	0 (0)
Number of cigarettes smoked per day (on smoking days), mean (SD; range)		10 (4.32; 2-15)
Smoking status of the partner (yes; n=25), n (%)		9 (36)
**Number of smokers in household, n (%)**		
	0	24 (59)
	1	14 (34)
	>2	3 (7)
Child exposure to indoor tobacco smoke (yes), n (%)		0 (0)
Child exposure to outdoor tobacco smoke (yes), n (%)		7 (17)
Child exposure to tobacco smoke in the car (yes), n (%)		0 (0)

^a^The Stolen Generations refers to a period in Australia’s history where Aboriginal children were removed from their families through government policies. This happened from the mid-1800s to the 1970s [33].

**Table 2:** Demographics of professionals (n=6).

Characteristic			Values
**Service type, n (%)**			
	Aboriginal medical service	3 (50)	
	Aboriginal preschool	3 (50)	
Sex (female), n (%)			6 (100)
**Indigenous status, n (%)**			
	Aboriginal	2 (33)	
	Torres Strait Islander	0 (0)	
	Nonidentified	4 (67)	
**Role at health service, n (%)**			
	Registered nurse	2 (33)	
	Midwife	1 (17)	
	Codirector or early educators	3 (50)	
Number of years at service, mean (SD; range)			10.5 (8.8; 1-25)

### User Engagement (n=41 Women)

#### App

Of the 41 women, 31 (76%) participants accessed the application. Among these 31 participants, there were 154 log-ins, with an average of 5 log-ins per person. Of these 31 women, 13 (42%) users scrolled the main page only, and the remaining 18 (58%) users moved past the main page by clicking on other pages. A total of 23% (7/31) of the users clicked on 10 website links.

A total of four user typologies were identified: (1) could not use the app, (2) obligated to use the app, (3) reviewers, and (4) researchers. The “couldn’t use the app” group included those who could not log into or download the application. The “obligated to use the app” group included those who used the app to provide feedback in a research context and probably would not use the application in a real-world setting. The “reviewer” group included those who logged in once or twice out of curiosity to see what the application included but did not consistently use the application. The “researcher” group included those who used the application more regularly; they were users who likely wanted more information than available in the SMS text messages or on the Facebook page. By analyzing the user activity metrics, we estimated that 20% (8/41) of the women were “researchers,” indicating that they may have long-term engagement with the application in the real world.

#### Videos

There were 48 plays of the 12 videos (Table 3). The number of plays ranged from 0 for “Milestones” and “Exercise” videos to 11 plays for “Sleeping” video. The number of unique viewers ranged from 1 to 5 per video. The highest number of unique viewers was for the “Feeling Good” video. Among the 48 plays, there were only 6 (13%) video completions. The mean viewing time was 38 seconds. The ability to obtain feedback on the video content may have been limited by the fact that the videos were not watched by most women. One reason for this was that to watch the videos on the Facebook page, the user needed to leave the Facebook page and watch them on an external host (Vimeo [Vimeo, Inc]). Another reason may have been that the videos were not clearly displayed in the application. Finally, the duration of the videos may have been too long.

**Table 3:** Engagement with the application videos.

Video	Plays (n=48), n (%)	Completions (n=6), n (%)	Viewing time (seconds)^a^, mean	Unique viewers, n^b^
Sleeping	11 (23)	1 (17)	22	3
Ear health	9 (19)	1 (17)	77	3
Eating well	7 (15)	1 (17)	6	3
Breastfeeding	5 (10)	1 (17)	115	4
Feeling good	5 (10)	0 (0)	63	5
Women’s business	4 (8)	1 (17)	72	1
Smoke free	4 (8)	0 (0)	11	1
Safe drinking	1 (2)	0 (0)	0	1
Vaccinations	1 (2)	0 (0)	0	1
Breathing well	1 (2)	1 (17)	89	1
Milestones	0 (0)	0 (0)	0	0
Exercise	0 (0)	0 (0)	0	0

^a^Total mean viewing time was 38 (SD 42.2) seconds.

^b^The average number of unique viewers was 2 (SD 1.62).

#### Facebook Page

Facebook administrators (Aboriginal researchers BH and NS) posted 101 posts over the 4-week pilot study. The page received 49 page likes and 51 followers, indicating reach beyond the study participants. The post with the highest reach was a supportive and affirming cultural post, which reached 308 people and had 17 reactions, comments, or shares. The second most popular post was a competition post, which reached 58 people and had 23 reactions, comments, or shares. The videos posted on Facebook (n=12) had an average of 20 people reach but only 1 to 2 reactions, comments or shares or clicks to watch externally.

#### SMS Text Messages

No participants opted out of the SMS text messages. The participants selected 3 topics to receive SMS text messages on. In the order of popularity, the topics chosen were ears, eyes, and teeth (23/41, 56%); sleeping (19/41, 46%); exercising (17/41, 41%); feeding and eating (13/41, 32%); eating (12/41, 30%); women’s business (12/41, 30%); milestones (10/41, 24%); feeling good (10/41, 24%); vaccinations and medicines (6/41, 14%); and safe drinking (0/41, 0%).

### Acceptability

#### Overview

**Figure 1:**
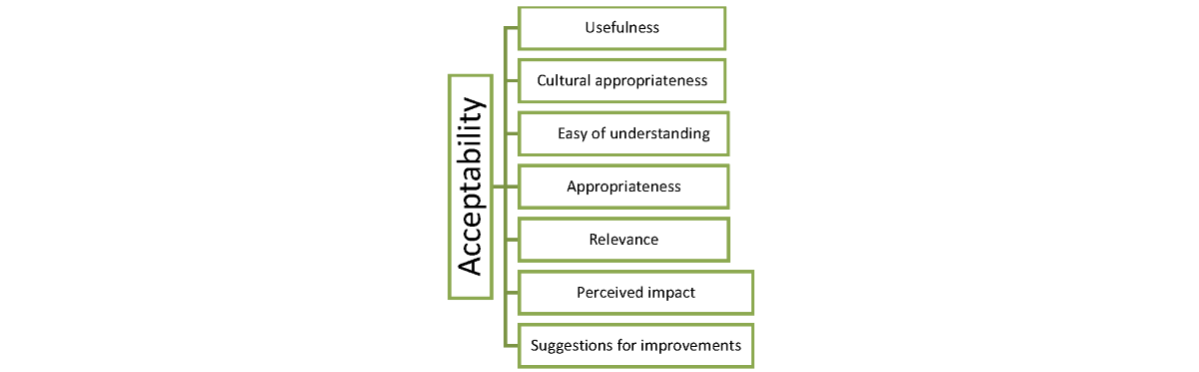
Themes.

Of the 41 women, 32 (78%) were interviewed at the end of the pilot study. All the 6 professionals were interviewed. There were 7 themes identified in the analysis related to the acceptability (Figure 1).

#### Usefulness

Almost all women (30/32, 94%) reported that the Growin’ Up Healthy Jarjums program was useful. On a scale of 1 (a little useful) to 5 (extremely useful), the mean rating of usefulness was 3.9. Furthermore, 84% (27/32) of the women reported that the program was relevant to them.

All women (30/32, 94%) reported that they would recommend the program to other families; the reasons included the following: helpful to first-time mothers, younger mothers, and mothers without family and other supports; ease of having all information in 1 spot; connection with other mothers; provides a sense of community; visually appealing and representative of Aboriginal and Torres Strait Islander people; and an accessible place for women feeling too ashamed or isolated to go the hospital or physician to access reliable health information:

I reckon just the feeling of still being connected, and being supported. I think it’s a nice way, especially for mums with little, little kids, I reckon sometimes you feel pretty isolated, especially if you’re not working and stuff. I think it’s a nice way to still feel like someone’s looking out for you or thinking of you.

Participant 6

I think it’s a good tool for our community, especially the young ones that we’ve got who may not have anywhere else to go to find that information or who are too ashamed to ask. I think having it in a way that they can find it themselves in an easy format is a good thing.

Participant 27

All professionals (6/6, 100%) reported that the Growin’ Up Healthy Jarjums program was useful. On a scale of 1 (a little useful) to 5 (extremely useful), the mean rating of usefulness was 3.3. All the professionals reported that they would recommend the program.

The professionals reported that the program would be useful to families for different reasons. Some of the cited reasons are as follows: the program is relatable to mothers because of co-design methods; using Facebook will result in family and friends seeing the health information; “Storytime” would be good for families that have less access to books; Facebook page was welcoming, easy to access, and visually attractive; the app was easy to navigate and would be easy for mothers to look through while “on the go”; overall, the program has a good balance of content, including health information, affirmations, and what is on in community; the Facebook page may be a good place for women to get ideas from each other and chat about recommendations from the posts; the Facebook page would be a good place for women to connect and not feel isolated; and the content is largely positive, which is important:

I like, on the Facebook page, it gives you a sense of not being so isolated. The Facebook page is a really nice place for Aboriginal mums or families to not feel isolated if they’re being recommended all this stuff and things to do outside of their lives, and then there’s the affirmations. It’s probably a really nice place for them to be especially if they’re suffering any mental health or with any isolation in their own lives.

Participant 6

#### Cultural Appropriateness

All the women (32/32, 100%) reported that the program was culturally appropriate. They reported that the colors, graphics, and language used were culturally representative. Of the 32 women, 1 (3%) woman recommended using different languages depending on what community the program is intended for. Another woman who had no exposure to her culture said that it was a helpful way to learn about her culture:

I’m Aboriginal, but I only just learnt of my Aboriginality, so I wasn’t actually brought up that way [according to her culture]. So it was helpful for me to learn new terminologies and stuff like that.

Participant 3

All the professionals reported that the program was culturally appropriate. A total of 33% (2/6) of the professionals commented on the cultural appropriateness of the language in a positive light. Overall, 17% (1/6) questioned whether the language would be difficult for some women to understand. She emphasized the need to ensure that representatives from each community that the program would be used in be involved in developing the content as well as to administer the Facebook page to ensure that the program continues to be relevant to women from different communities:

You’ve got different lingos, different meanings, different sayings that’s going to grab attention [in different communities]. When I look through these text messages there are some words in here that I would think some of our women wouldn’t really understand. You actually need the women to do it, they’re very different to the workers. They’re the ones that are going to give you the right language.

Participant 1

It’s all culturally appropriate...the language is really nice. It’s really easy to read and interpret. It doesn’t have any of those big, yukky words that can be quite clinical.

Professional 5

#### Easy to Understand

All the women (32/32, 100%) reported that the content was easy to understand. They talked about the program being jargon free but not too simplified:

It was spoken to you normally, not like all the medical jargon, do you know what I mean? It was understandable and relatable. I think if it’s too technical sometimes, it gets overwhelming.

Participant 20

It wasn’t dumbed-down. Like some things that we give to Aboriginal families that we get, some of them are so simplified that makes people think that we’re stupid, but this wasn’t. It was easy to understand, but it didn’t make me feel stupid, didn’t make me feel bad.

Participant 27

#### Appropriate

Most women (28/32, 88%) reported that the activities and information were appropriate. However, of the 32 women, 1 (3%) woman suggested that the language in the SMS text messages could have been more professional rather than colloquial:

We followed what was on those messages each day. We made it a project with our kids because we thought we would see how it would go. My son likes looking at the pictures of it when they’ve had the Facebook competitions because it was all kids that he knew.

Participant 27

#### Relevance

Most women (27/32, 84%) reported that the program was relevant to them and their family. They said that it was good having other mothers to relate to and access to reliable health information to talk to family about:

Just sort of seeing other women with kids and stuff and sort of just having someone to relate to, somebody that’s a little bit more similar to me. So you don’t feel so alone in what you’re kind of going through. Things that you might think are silly or you’re a bit shamed to ask anyone. At least the messages address it and then you can see other women on the Facebook page.

Participant 4

I learnt some good stuff. I think the thing I like most about the program for us personally, it allowed me to have conversations with my partner about smoking. With the program I was able to say ‘hey I got a text about smoking,’ ‘do you know this’ it meant that it wasn’t just me making points. I sort of used the app as the conversation starter.

Participant 18

The women who did not find the content relevant to them (2/32, 6%) said that they already knew the information or felt that the information was targeted toward women caring for younger children. Of the 32 women, 2 (6%) women also made comments about some SMS text messages not always being relevant to their family:

Some of them [SMS] might not be relevant to me personally, but I think as a community they are. The messaging was consistent, and I think that’s really important.

Participant 6

My little boy is older now, he’s two. So I felt like a lot of the messages and stuff like that, was more around the newborn stuff. But in saying that, if I had a newborn still, then it would have been more relevant.

Participant 10

#### Perceived Impact

Of the 31 women who commented on the overall positive impact of the program, 22 (71%) women reported that the program had an overall positive impact on themselves and their family. Table 4 presents a summary of the perceived impacts. The most common perceived impact was feeling more supported (21/29, 72%), followed by improvements in knowledge or understanding of child health (13/26, 50%), eating habits (11/29, 38%), and exercise (11/29, 38%). Many women commented on the supportive and affirming aspects that the program provided, including the validation of how hard parenting can be; information that certain health problems, such as ear infections, are common in children (validating that it was not their fault); the feeling that a service cared about their child’s health; and the feeling that someone cared about them, which arose because of the reception of regular messages:

The favourite text of mine was the reminder that we all have rough days...And that it was okay, I thought you know what, yeah, I am going to take a breath right now, and it is all okay.

Participant 10

It was good to know that there was a service out there that did care I guess or had an interest in my son’s health.

Participant 14

Other positive impacts that the women discussed included getting their child’s ear health checked by a general practitioner, more play with children, spending time together as a family, taking children for hearing and vision tests, more exercise, taking care of themselves, talking to friends and family about quitting or reducing smoking, cooking with children, limiting alcohol, getting their child immunized, improved knowledge of contraception, and improved family eating:

Usually I’m the type where I walk to the park and then watch her play. When I read the messages, I’m like, I should actually try with her more, and be more active with her at the park.

Participant 24

I was drinking far too much, it was just a stress handling thing, because we did have a lot of problems, and it was difficult the first couple years. And so, getting that information, that really helped me to kind of kick that habit and to look at my own lifestyle and stuff.

Participant 9

Other women experienced stressful life situations at the time and had competing priorities, limiting the potential impacts of the program.

**Table 4:** Perceived impact (n=32).

	Participants who responded, n (%)	Participants whose response was yes, n (%)
Overall positive impact	31 (97)	22 (71)
Improvements to your smoking habits (if a smoker)	30 (94)	5 (17)
Family or friend smoking habits (if a smoker)	30 (94)	2 (7)
Child’s exposure to second-hand smoke	29 (91)	8 (28)
Positive impact on family eating habits	29 (91)	11 (38)
Positive impact on physical activity	29 (91)	11 (38)
Improvements to knowledge of women’s health	28 (88)	8 (29)
Improvements to knowledge of child health	26 (81)	13 (50)
Feeling more supported	29 (91)	21 (72)

#### Suggestions for Improvements

The most common suggestion for improving the program was to make changes to the application to overcome technical challenges. Of 32 women, at least 6 (19%) experienced technical problems. Difficulty in downloading and saving the web-based application, rather than accessing an Android or iOS (Apple Inc) app, was the main difficulty. Almost half of the women (14/32, 44%) suggested improvements to the application, including making the web-based application an Android or iOS app so that it “looks” like an app, removing the step of saving to the application to home screen, implementing single log in, making navigation simpler, making the application accessible on all phone types, localizing the application to specific communities, and making the application more interactive:

I had to log back in and it would take me to the web page. It kept wanting me to resave it to my home screen, but it was already saved to my home screen. I wasn’t too sure what was going on there.

Participant 30

The next most common suggestion is related to the SMS text messages. A total of 59% (19/32) of the women reported that there was just the right number of SMS text messages, but 38% (12/32) of the women reported that the SMS text messages were too frequent. The women indicated the preferred timing of SMS text messages to be earlier in the day and a preference to have SMS text messages from the same sender phone number for ease of reviewing.

Other suggestions to improve the program overall included suggestion to ensure better tailoring to the child’s age as well as suggestions for alternate topics (eg, parenting, toilet training, separation, toddler development, mental development for boys, available services, preschool readiness, allergies, and resources and services specific to Aboriginal people), suggestion to provide less content about smoking, suggestion to provide more links to further information (eg, local mothers’ groups), and suggestion to provide to make the program more interactive:

If it is targeting under five, some of the information could be more around toilet training, difficulties with separation, entry to preschool, advice on services and stuff, like Koori stuff around.

Participant 9

In total, 6% (2/32) of the women talked about web-based groups: 1 woman suggested a Facebook group (rather than a Facebook page) and another suggested a chat group within the application (to discuss specific topics). Moreover, 6% (2/32) of other women talked about the importance of the continuation of the program and the longevity of Aboriginal and Torres Strait Islander health programs in general, as Aboriginal health programs often have short funding cycles and the community is left with a gap:

I would have benefited more with the Facebook group if it was an actual group created, because normally you get notifications and stuff when you’re in the group and it tells you who’s posted what. I probably would have had more interaction with that if that was a constant notification coming up.

Participant 13

Keep it going. We find sometimes that programs are really good and then they stop. When the funding runs out or it doesn’t get approved or whatever it stops and then that’s a gap.

Participant 27

The professionals suggested several ways to improve the program, including continued involvement of women in the development of the program to ensure that the language and content remain relevant and appropriate, including for families living in regional and remote areas. Another professional suggested that the content should be current and tailored to the age of the child. Another suggested a “search” feature in the application so that families could easily search for the health issue or topic that they are interested in (otherwise, mothers will likely Google health information, which can make it difficult to determine reliable sources). Another suggested more specific steps about how to manage certain illnesses and to include more common childhood illnesses. Another suggested that the application needs to be more interactive, for example, tailored specifically to the child’s age, with notifications for activities for that age group or milestones. One other professional suggested forums or private group chats so that discussions are not public, whereas another suggested providing grandparents and other family members access to the program.

## Discussion

### Principal Findings

Overall, the Growin’ Up Healthy Jarjums program was found to have high acceptability. The results indicate that women found the program to be useful, culturally appropriate, and easy to use, and most women reported positive impacts. None of the participants withdrew from the SMS text message portion of the program, which indicated the high acceptability of and engagement with this component. Engagement with the Facebook page was found to be higher than that with the application. Individual users preferred different modes (SMS text message, Facebook page, or application), indicating that a multimodal intervention increases reach. Importantly, this pilot study showed several ways to improve the program, including technical changes to the app.

Similar to other studies with Aboriginal and Torres Strait Islander people, the SMS text message component of this program appeared to have high acceptability and engagement [12-14]. This pilot study provided an additional opportunity to focus on mothers and examine the desired frequency of SMS text messages. The frequency of SMS text messages sent in mHealth trials is often 1 SMS text message per day, although it can vary from multiple SMS text messages per day to weekly SMS text messages [34]. In our study, women were sent 1 SMS text message per day for 5 days of the week over 4 weeks. Most women (19/32, 59%) reported that the frequency was just right, 38% (12/32) of the women reported that the SMS text messages were sent too often, and 3% (1/32) of the women reported that the SMS text messages sent were not enough. This finding is similar to that of another study that reported that 1 SMS text message per day was preferred by the majority (42%), whereas the remaining 58% preferred either more or less frequency [24]. Giving a choice of frequency of either 1 SMS text message per day or 3 SMS text messages per week may be more appealing to users.

The women reported that they liked having a choice of topics for SMS text messages, as this increased relevance. Some women commented on the core (requisite) topics, indicating (1) breathing well and (2) smoke-free families as being irrelevant to them and their families. SMS text messages on smoking cessation and child lung health were requisite based on the original focus of the intervention to promote child lung health, including smoking cessation. The focus on child lung health was based on the call for more culturally appropriate information on childhood coughs [35]. In addition, there is strong evidence that SMS text messages are effective for quitting smoking [36]; thus, we decided to keep the focus on child lung health, including smoking cessation, for the SMS text message portion of the pilot. Furthermore, many of the SMS text messages were targeting the first 2 years of life, as that was the age range in which we expected to recruit most children; however, many of the children were older, which meant that some of the information was not relevant, although most women commented that they could see how beneficial the information would have been when their children were young. Encouragingly, the number of women who reported the SMS text messages to be irrelevant was low (3/30, 10%), similar to another study on SMS text messages for new mothers (6/22, 21%) [31]; however, giving users the choice of all topics may be a more acceptable and useful approach allowing for better tailoring to end users’ health information needs and interests.

Many participants experienced technical challenges in accessing the app, with nearly one-fourth (10/41, 24%) having been unable to access it. The prototype application used in the pilot study was a web-based application. A web-based application is accessed through an internet browser, such as Google Chrome (Google LLC) or Firefox (Mozilla Foundation), and is essentially a website designed to look like an Android or iOS app. Android or iOS apps are downloaded from an app store and saved on the phone [37]. The benefits of web-based applications are that they are fast to build, they are cost-effective, and their content can be changed easily [37]. The benefits of Android or iOS apps are that they are faster than web-based applications; they can work without internet connection; and end users are generally more familiar with them, including with downloading and saving them [37]. Using a web-based application for this trial resulted in many women having difficulty logging in and saving the application on their home screen. The women also commented that it did not “look” like an application and that it was slow. Other application trials with Aboriginal and Torres Strait Islander people have largely used Android or iOS apps [15,38-41]. Of these studies, 1 was unable to collect use data for 34% (21/61) of the participants reporting flat batteries, connectivity issues, and other problems [41]. A second study reported technical difficulties for participants with using the “challenge” function and signing in and out, although it was noted that technical challenges did not significantly impact the use for many participants [15]. In a third study, an app was used by clients with a practitioner present, and technical difficulties were reported with an Android emulator to enable compatibility with Windows [40]. The fourth study used the same app as the previous study [40] with a different population; thus, it was also used by clients with a practitioner present, but no technical difficulties were reported [39]. Although many studies evaluating mHealth apps have reported technical challenges, it seems that using a web-based application may have resulted in more users experiencing technical challenges and a more substantial challenge of not being able to log in or save the application. With a large proportion of women having had difficulty accessing the application, most of the feedback on the application was centered on the technical challenges, and there was limited feedback on the content. However, it was useful to discover during this early phase that a web-based application is not feasible. An Android or iOS app will need to be considered before further evaluation with a small group of end users to provide detailed feedback on content and navigation.

In addition to considering the technical barriers to accessing applications, careful consideration must be given to long-term engagement [16]. In a longitudinal study examining the reasons for continued use of mHealth apps, two connected factors were described: (1) users’ assessment of the mHealth app (related to the technology and content) and (2) users’ persistence of health goals (ie, those who have higher persistence toward reaching their goals appear to have longer engagement with health apps) [16]. The authors concluded that long-term engagement with health apps occurs when there is high user assessment and high persistence toward health goals [16]. With improvements to the technical aspects of the Growin’ Up Healthy Jarjums application, we expect to see improvements in initial access and a small increase in long-term engagement, although it is evident that an application is not going to be engaging to all users. Health applications are suggested to be most engaging for users who are younger, are more educated, and have higher levels of eHealth literacy skills [42]. It is also suggested that the use of health applications can improve when support from a clinician or another medical professional is provided [43]. The findings from this pilot would also suggest that those experiencing distressing life situations may find it difficult to engage with a health application, similar to the findings that suggest mental health applications may be more suitable for those with less severe illness [43]. As health applications are likely to continue to improve as technology continually does, engagement with health applications will also improve. However, at this point in time, it seems apparent that mHealth tools should be provided in a range of delivery modes to increase reach, digital inclusivity, and equity.

One such delivery mode is social media. It has been established that Aboriginal and Torres Strait Islander people are avid users of social media, Facebook in particular [8-11], which is a key reason why a Facebook page was part of the Growin’ Up Healthy Jarjums program. The qualitative findings from our pilot suggest that the Facebook page had high acceptability. The women commonly reported that they valued the connection and seeing what other families were doing. It can be difficult to track engagement with Facebook using objective data because of privacy measures and the complexity of identifying whether users accessed the page as “observers,” rather than more active users, which can be done only by examining page likes, comments, shares, etc [10]. A qualitative study examining social media and health information sharing among Aboriginal and Torres Strait Islander people shed more light on how social media are used for health promotion by identifying six typologies: (1) observer, (2) post sharer, (3) positive supporter, (4) educator, (5) expert, and (6) influencer [10]. Although we do not have the data to compare all typologies with the previous study, our results indicate that mothers were more likely to be “observers,” with many women reporting the value of connection and seeing what other families were doing but not often commenting, liking, or sharing posts during the study. Posts that were shared or commented on were more likely to have been posts uploaded by Aboriginal organizations, posts affirming Aboriginal culture, or posts about competitions where a prize could be won. The previous study used a methodology different from the one used in this study, wherein they had participants monitor their social media accounts for health-related content and conducted weekly interviews to explore perspectives and actions on posts. Interestingly, the authors found that users moved between typologies depending on the health topic and how information was provided [10]. In future research on the Growin’ Up Healthy Jarjums Facebook page, it may be useful to use a similar methodology with a subset of participants to better understand what and how health information is shared among mothers with young children, as well as how this correlates with changes in health literacy offline; given the high acceptability of and engagement with Facebook among this group of end users, Facebook has great potential to improve health literacy.

### Limitations

A limitation of this study was the need to conduct all recruitment and instruction of the program remotely using SMS text messages, links, and phone calls owing to COVID-19 restrictions. In the initial protocol, we proposed recruiting women, setting up the app, and providing instructions on how to use the application in person to reduce technical problems. Unfortunately, this was not possible, and women experienced a high number of technical problems. With a large proportion of women having had difficulty accessing the application, most of the feedback on the application was centered on the technical challenges, and there was limited feedback on the content; however, it was useful to discover during this early phase that a web-based application is not feasible. An initial in-person setup would be considered important for further use of the program.

Another limitation of this study is that generalization to other communities is limited. Aboriginal and Torres Strait Islander communities are made up of many diverse cultural and language groups [1]. Each community has a unique history, cultural practices, and health needs. The Growin’ Up Healthy Jarjums program would need to be adapted, including by making changes to language, images, and health advice, to ensure cultural safety and relevance to women from other communities.

### Conclusions

This study demonstrates that the Growin’ Up Healthy Jarjums program was perceived as useful and culturally appropriate by users and health professionals. The SMS text messages had the highest engagement, followed by the Facebook page and then the application. This study identified suggestions for improving the application. A trial is needed to assess the effectiveness of the Growin’ Up Healthy Jarjums program at improving health outcomes.

## Abbreviations

AH&MRC: Aboriginal Health and Medical Research Council
mHealth: mobile health
NSW: New South Wales
REDCap: Research Electronic Data Capture

## References

[1] Explore. Australian Institute of Aboriginal and Torres Strait Islander Studies.

[2] Stolen Generations. Healing Foundation.

[3] National aboriginal and Torres strait islander health plan 2013–2023. Australian Government Department of Health and Aged Care.

[4] Improving health literacy. World Health Organization.

[5] Nash S, Arora A (2021). Interventions to improve health literacy among Aboriginal and Torres Strait Islander Peoples: a systematic review. BMC Public Health.

[6] Lupton D (2019). The Australian Women and Digital Health Project: Comprehensive Report of Findings.

[7] Perkes SJ, Bonevski B, Hall K, Mattes J, Chamberlain C, Bennett J, Whittaker R, Palazzi K, Lambkin D, Kennedy M (2023). Aboriginal and Torres Strait Islander women’s access to and interest in mHealth: national web-based cross-sectional survey. J Med Internet Res.

[8] Carlson B, Frazer R (2018). Social Media Mob: Being Indigenous Online.

[9] McPhail-Bell K, Appo N, Haymes A, Bond C, Brough M, Fredericks B (2018). Deadly Choices empowering Indigenous Australians through social networking sites. Health Promot Int.

[10] Hefler M, Kerrigan V, Henryks J, Freeman B, Thomas D (2019). Social media and health information sharing among Australian Indigenous people. Health Promot Int.

[11] Rice ES, Haynes E, Royce P, Thompson SC (2016). Social media and digital technology use among Indigenous young people in Australia: a literature review. Int J Equity Health.

[12] Kirkham R, MacKay D, Barzi F, Whitbread C, Kirkwood M, Graham S, Van Dokkum P, McIntyre HD, Shaw JE, Brown A, O'Dea K, Connors C, Oats J, Zimmet P, Boyle J, Maple‐Brown L (2018). Improving postpartum screening after diabetes in pregnancy: results of a pilot study in remote Australia. Aust N Z J Obstet Gynaecol.

[13] Phillips JH, Wigger C, Beissbarth J, McCallum GB, Leach A, Morris PS (2014). Can mobile phone multimedia messages and text messages improve clinic attendance for Aboriginal children with chronic otitis media? A randomised controlled trial. J Paediatr Child Health.

[14] (2014). Media usage amongst Aboriginal and Torres Strait Islander people. McNair Ingenuity Research.

[15] Peiris D, Wright L, News M, Rogers K, Redfern J, Chow C, Thomas D (2019). A smartphone app to assist smoking cessation among aboriginal Australians: findings from a pilot randomized controlled trial. JMIR Mhealth Uhealth.

[16] Vaghefi I, Tulu B (2019). The continued use of mobile health apps: insights from a longitudinal study. JMIR Mhealth Uhealth.

[17] National best practice unit tackling Indigenous smoking. Ninti One Limited.

[18] Houston A, Laws R, Askew D, Saldanha T, Denney-Wilson E (2017). Exploring the cultural appropriateness and usefulness of a mHealth promotion program for infant feeding in an Urban Aboriginal Health Service: a qualitative study. Aust Indig Health Bull.

[19] Kennedy M, Kumar R, Ryan NM, Bennett J, La Hera Fuentes G, Gould GS (2021). Codeveloping a multibehavioural mobile phone app to enhance social and emotional well-being and reduce health risks among Aboriginal and Torres Strait Islander women during preconception and pregnancy: a three-phased mixed-methods study. BMJ Open.

[20] ANMJ Staff (2019). Mindfulness app improves mental health in Indigenous communities. Australian Nursing and Midwifery Journal.

[21] Deadly tots app. Deadly Tots.

[22] Yarn and heal - our way. Facebook.

[23] Perkes SJ, Huntriss B, Skinner N, Leece B, Dobson R, Mattes J, Hall K, Bonevski B (2022). Development of a maternal and child mHealth intervention with aboriginal and torres strait islander mothers: co-design approach. JMIR Form Res.

[24] Whittaker R, Merry S, Dorey E, Maddison R (2012). A development and evaluation process for mHealth interventions: examples from New Zealand. J Health Commun.

[25] Michie S, Yardley L, West R, Patrick K, Greaves F (2017). Developing and evaluating digital interventions to promote behavior change in health and health care: recommendations resulting from an international workshop. J Med Internet Res.

[26] AH and MRC Ethical Guidelines: Key Principles (2020) V2.0. Aboriginal Health and Medical Research Council of New South Wales.

[27] Parker C, Scott S, Geddes A (2019). Snowball sampling. Research Design for Qualitative Research.

[28] Creswell J (2009). Qualitative procedures. Research Design: Qualitative, Quantitative, and Mixed Methods Approaches.

[29] Hall KK, Chang AB, Anderson J, Arnold D, Goyal V, Dunbar M, Otim M, O’Grady KF (2017). The incidence and short-term outcomes of acute respiratory illness with cough in children from a socioeconomically disadvantaged urban community in Australia: a community-based prospective cohort study. Front Pediatr.

[30] Ng MM, Firth J, Minen M, Torous J (2019). User engagement in mental health apps: a review of measurement, reporting, and validity. Psychiatr Serv.

[31] Dobson R, Whittaker R, Bartley H, Connor A, Chen R, Ross M, McCool J (2017). Development of a culturally tailored text message maternal health program: TextMATCH. JMIR Mhealth Uhealth.

[32] Creswell J (2018). Quantitative methods. Research Design: Qualitative, Quantitative, and Mixed Methods Approaches.

[33] Who are the stolen generations?. Healing Foundation.

[34] Hall AK, Cole-Lewis H, Bernhardt JM (2015). Mobile text messaging for health: a systematic review of reviews. Annu Rev Public Health.

[35] D'Sylva P, Walker R, Lane M, Chang AB, Schultz A (2018). Chronic wet cough in Aboriginal children: it's not just a cough. J Paediatr Child Health.

[36] Whittaker R, McRobbie H, Bullen C, Borland R, Rodgers A, Gu Y (2012). Mobile phone-based interventions for smoking cessation. Cochrane Database Syst Rev.

[37] Difference between native apps and web apps. Geeks for Geeks.

[38] Tighe J, Shand F, Ridani R, Mackinnon A, De La Mata N, Christensen H (2017). Ibobbly mobile health intervention for suicide prevention in Australian Indigenous youth: a pilot randomised controlled trial. BMJ Open.

[39] Nagel T, Dingwall KM, Sweet M, Kavanagh D, Majoni SW, Sajiv C, Cass A (2022). The stay strong app as a self-management tool for first nations people with chronic kidney disease: a qualitative study. BMC Nephrol.

[40] Perdacher E, Kavanagh D, Sheffield J, Healy K, Dale P, Heffernan E (2022). Using the stay strong app for the well-being of Indigenous Australian prisoners: feasibility study. JMIR Form Res.

[41] Tighe J, Shand F, McKay K, Mcalister T, Mackinnon A, Christensen H (2020). Usage and acceptability of the iBobbly app: pilot trial for suicide prevention in Aboriginal and Torres Strait Islander Youth. JMIR Ment Health.

[42] Bol N, Helberger N, Weert JC (2018). Differences in mobile health app use: a source of new digital inequalities?. Inform Society.

[43] Povey J, Mills PP, Dingwall KM, Lowell A, Singer J, Rotumah D, Bennett-Levy J, Nagel T (2016). Acceptability of mental health apps for Aboriginal and Torres Strait Islander Australians: a qualitative study. J Med Internet Res.

